# An ANOCEF genomic and transcriptomic microarray study of the response to radiotherapy or to alkylating first-line chemotherapy in glioblastoma patients

**DOI:** 10.1186/1476-4598-9-234

**Published:** 2010-09-07

**Authors:** François Ducray, Aurélien de Reyniès, Olivier Chinot, Ahmed Idbaih, Dominique Figarella-Branger, Carole Colin, Lucie Karayan-Tapon, Hervé Chneiweiss, Michel Wager, François Vallette, Yannick Marie, David Rickman, Emilie Thomas, Jean-Yves Delattre, Jérôme Honnorat, Marc Sanson, François Berger

**Affiliations:** 1 INSERM, U842, Lyon, F-69372 France; Université Lyon 1, UMR-S842 Lyon, F-69003 France; 2 Programme Cartes d'Identité des Tumeurs (CIT), Ligue Nationale Contre le Cancer, Paris, France; 3 Université de la Méditerranée, Faculté de Médecine de Marseille, Assistance Publique-Hôpitaux de Marseille, Unité de Neuro-Oncologie, Centre Hospitalier Universitaire Timone, 264 rue Saint Pierre, 13385 Marseille Cedex 05, France; 4 Hôpital de la Salpêtrière (APHP), INSERM U711 and Université P&M Curie, Paris, France; 5 « Equipe Angiogenèse, Invasivité et Microenvironnement tumoral » Faculté Médecine Timone, Université de la Mediterrannée UMR911 CRO2, Service d'Anatomie Pathologique et de Neuropathologie, Assistance Publique des Hôpitaux de Marseille, hôpital de la Timone, Bd Jean Moulin 13385 Marseille cedex 05, France; 6 Université de Poitiers, EA3805, CHU de Poitiers, 86022 Poitiers cedex, France; 7 UMR 894 INSERM, Faculté de Médecine Université Paris Descartes, Paris, France; 8 Centre de Recherche en Cancérologie Nantes Angers, Centre INSERM U892, Université de Nantes, 9 quai Moncousu 44035 Nantes cedex 01 France; 9 Inserm U836, Grenoble Institut de Neurosciences, Unité Joseph Fourier, 38042 Grenoble Cedex 9, France; 10 ANOCEF (Association des Neuro-Oncologues d'Expression Française -French Speaking NeuroOncologists' Association), Unité de neuro-oncologie CHU Timone 264, rue Saint Pierre 13385 Marseille Cedex 5

## Abstract

**Background:**

The molecular characteristics associated with the response to treatment in glioblastomas (GBMs) remain largely unknown. We performed a retrospective study to assess the genomic characteristics associated with the response of GBMs to either first-line chemotherapy or radiation therapy. The gene expression (n = 56) and genomic profiles (n = 67) of responders and non-responders to first-line chemotherapy or radiation therapy alone were compared on Affymetrix Plus 2 gene expression arrays and BAC CGH arrays.

**Results:**

According to Verhaak et al.'s classification system, mesenchymal GBMs were more likely to respond to radiotherapy than to first-line chemotherapy, whereas classical GBMs were more likely to respond to first-line chemotherapy than to radiotherapy. In patients treated with radiation therapy alone, the response was associated with differential expression of microenvironment-associated genes; the expression of hypoxia-related genes was associated with short-term progression-free survival (< 5 months), whereas the expression of immune genes was associated with prolonged progression-free survival (> 10 months). Consistently, infiltration of the tumor by both CD3 and CD68 cells was significantly more frequent in responders to radiotherapy than in non-responders. In patients treated with first-line chemotherapy, the expression of stem-cell genes was associated with resistance to chemotherapy, and there was a significant association between response to treatment and *p16 *locus deletions. Consistently, in an independent data set of patients treated with either radiotherapy alone or with both radiotherapy and adjuvant chemotherapy, we found that patients with the *p16 *deletion benefited from adjuvant chemotherapy regardless of their *MGMT *promoter methylation status, whereas in patients without the *p16 *deletion, this benefit was only observed in patients with a methylated *MGMT *promoter.

**Conclusion:**

Differential expression of microenvironment genes and *p16 *locus deletion are associated with responses to radiation therapy and to first-line chemotherapy, respectively, in GBM. Recently identified transcriptomic subgroups of GBMs seem to respond differently to radiotherapy and to first-line chemotherapy.

## Background

Microarrays are an effective tool for the study of glioma oncogenesis, and this technique has enabled the discovery of new molecular pathways implicated in gliomagenesis [1,2]. Several studies have also described molecular signatures related to histological type and to survival in high-grade gliomas [3-10]. However, until now, few studies have used microarray technology to elucidate the mechanisms associated with the response of the tumor to treatment [11,12]. Despite an overall grim prognosis, some patients with glioblastoma (GBM) do respond to radiotherapy and chemotherapy and achieve prolonged survival. The molecular characteristics associated with prolonged progression-free survival (PFS) after radiotherapy in GBM patients remain largely unknown. Studies demonstrate that patients with methylation of the O6-methylguanine methyltransferase promoter (MGMTP) benefit from radiotherapy with concomitant temozolomide and from adjuvant chemotherapy with alkylating agents [13,14]. However, it is likely that this it is not the only mechanism underlying the chemosensitivity of the tumor in these patients. In this study, we examined the molecular characteristics associated with a response to radiotherapy or to first-line chemotherapy in GBMs in a cohort of patients treated with radiation therapy alone or with first-line chemotherapy; the genomic and transcriptomic profiles of responders and non-responders were compared for both treatment groups.

## Methods

### Patients

All patients included in this study had de novo GBM according to the 2007 World Health Organization Classification. A central pathological review was performed by DFB. In order to focus more specifically on treatment response, progression-free survival (PFS) and MacDonald's criteria of response were used as outcome measures rather than overall survival (OS), which may be influenced by the use of salvage treatment at relapse. Response to radiation therapy was defined in terms of PFS. Patients were considered as responders to radiotherapy if the PFS was >10 months and as non-responders if the PFS was <5 months. Patients treated with radiotherapy and concomitant temozolomide were excluded. In patients treated with first-line chemotherapy, response was evaluated before radiotherapy according to MacDonald's criteria, and all of these patients had an evaluable tumor [15]. These patients were treated with alkylating agents (BCNU or temozolomide). Radiotherapy was administered at progression or after six months of chemotherapy. Patients were considered as responders if they achieved either partial or complete response and as non-responders if they progressed during chemotherapy. Patients' clinical characteristics are available in Additional file 1 Table S1.

### Samples

Samples were provided as snap-frozen sections of areas immediately adjacent to the region used for the histopathological diagnosis. Only samples representative of the tumor and from which high-quality DNA and/or RNA could be obtained were selected (n = 86). For the comparative genomic hybridization (CGH) array study, 67 samples were available: 21 responders to radiotherapy, 18 non-responders to radiotherapy, 11 responders to first-line chemotherapy and 17 non-responders to first-line chemotherapy. The gene expression array study was performed on 56 samples (including 37 samples common to the CGH study): 19 responders to radiotherapy, 15 non-responders to radiotherapy, 12 responders to first-line chemotherapy and 10 non-responders to first-line chemotherapy.

### DNA extraction and hybridization

#### DNA was extracted from frozen tumors using a standard phenol-chloroform procedure

After digestion with DpnII (Ozyme, Saint Quentin en Yvelines, France) and column purification (Qiaquick PCR purification kit; Qiagen, Courtaboeuf, France), tumor DNA was labeled with cyanine-5 (Perkin-Elmer, Wellesley, MA) using the random priming method (Bioprime DNA labeling system; Invitrogen, Cergy-Pontoise, France). Using the same procedure, we labeled control DNA with cyanine-3. After ethanol co-precipitation with 210 g of human Cot-1 DNA (Invitrogen, Cergy-Pontoise, France), resuspension in hybridization buffer (50% formamide), denaturation at 95°C for 10 minutes and prehybridization at 37°C for 90 minutes, probes were cohybridized on an aCGH slide. The aCGH slide was previously preblocked with a buffer containing 2.6 mg succinic anhydride, 118 ml *N*-methyl-2-pyrrolidinone and 32 ml sodium tetraborate decahydrate, pH 8.0 (Sigma-Aldrich, Lyon, France). After washing, arrays were scanned using an Agilent 2565BA scanner. Image analysis was performed with SPOT v.2.1cc software, and the ratios of Cy5/Cy3 signals were determined. The human genome-wide CIT-CGHarray (V6), which contains 4,434 sequence-verified bacterial artificial chromosome (BAC) and P1-derived artificial chromosome clones, was chosen to obtain systematic coverage of the genome and detailed coverage of regions containing genes previously implicated in carcinogenesis. This array was designed by the CIT-CGH consortium (Olivier Delattre laboratory, Curie Institute, Paris; Charles Theillet laboratory, CRLC Val d'Aurelle, Montpellier; Stanislas du Manoir laboratory, IGBMC, Strasbourg) and IntegraGen. All clones were spotted in quadruplicate (and spaced at approximately 670 kb intervals) on Ultra Gaps slides (Corning Inc., Corning, NY).

### RNA extraction and hybridization

Approximately 50 mg of tissue from each tumor was used for total RNA extraction using the RNeasy Lipid Tissue mini kit (Qiagen, CA) according to the manufacturer's instructions. RNA quality was verified with the Bioanalyzer System (Agilent Technologies, Palo Alto, CA) using the RNA Nano Chip. RNA (1.5 μg) was processed and hybridized to the Genechip Human Genome U133 Plus 2.0 Expression array (Affymetrix, CA), which contains over 54,000 probe sets analyzing the expression levels of over 47,000 transcripts and variants. This roughly corresponds to 29,500 distinct Unigene identifiers. The processing was done according to the recommendations of the manufacturer.

### Immunohistochemistry

Immunohistochemistry was performed on tissue microarrays (TMA) comprising 25 GBMs (15 responders and 10 non-responders to radiotherapy for whom enough material was available) that were constructed from routinely processed formalin-fixed paraffin-embedded tumor material. Areas of viable and representative tumor, as determined by a review of all blocks, were marked by a pathologist (DFB) prior to inclusion in the TMA (3 × 0.6-mm cores for each tumor).

After steam-heat-induced antigen retrieval, 5-μm sections of formalin-fixed paraffin-embedded samples were tested for the presence of CD3, CD20 and CD68 using a polyclonal rabbit antibody (1:2) (Dako, Trappes, France), a monoclonal mouse antibody (1:600, L26) (Dako) and a monoclonal mouse antibody (1:5000, KP1) (Dako), respectively. A Benchmark Ventana autostainer (Ventana Medical Systems SA, Illkirch, France) was used for detection, and TMA slides were simultaneously immunostained to avoid inter-manipulation variability. Immunostaining was scored by a pathologist (DFB) as follows: 0 = no positive cell; + = some positive cells; ++ = a clear CD3, CD20 or CD68 infiltration.

### MGMT promoter methylation status

The MGMT promoter's (MGMTP) methylation status was assessed in patients treated with first-line chemotherapy. The DNA methylation status of the MGMT promoter was determined by bisulfite modification and subsequent Nested Methylation Specific PCR as previously described [16]. Sodium bisulfite specifically modifies non-methylated cytosines, but not methylated cytosines, to uracil. The sodium bisulfite treatment was carried out using the EZ DNA Methylation Kit (Zymo Research). The stage-1 PCR amplifies a 289-bp fragment of the MGMT gene using primers that do not discriminate between methylated and unmethylated alleles. The primer sequences are as follows: Forward 5′ GGATATGTTGGGATAGTT 3′, Reverse 5′ CCAAAAACCCCAAACCC 3′. PCR conditions were as follows: 95°C for 15 min, then 30 cycles of 95°C for 30 s, 52°C for 30 s and 72°C for 30 s, and finally 10 min at 72°C. The stage-1 PCR products were then diluted 100-fold, and 5 μl was subjected to a stage-2 PCR in which primers were specific to methylated or unmethylated alleles. Primer sequences are as follows: for the methylated reactions, Forward 5′ TTTCGACGTTCGTAGGTTTTCG 3′, Reverse 5′ GCACTCTTCCGAAAACGAAACG 3′; for the unmethylated reactions, Forward 5′ TTTGTGTTTTGATGTTTGTAGGTTTTTGT 3′, Reverse 5′ AACTCCACACTCTTCCAAAAACAAAACA 3′. PCR conditions were as follows: 95°C for 15 min, then 30 cycles of 95°C for 30 s, 62°C for 30 s and 72°C for 30 s, and finally 10 min at 72°C. PCR products were separated on 2% agarose gels stained with ethidium bromide and visualized under UV illumination. As a positive control for methylated alleles, we used DNA from lymphocytes treated with SssI methyltransferase (New England Biolabs: Ozyme, St-Quentin-Yvelines, France) and modified by bisulfite treatment.

### Data analysis summary

All raw and normalized data files for the microarray analysis have been deposited under accessions E-TABM-897 and E-TABM-898 at the European Bioinformatics Institute http://www.ebi.ac.uk/microarray-as/ae. All genomic and transcriptomic analysis was carried out using R software http://www.R-project.org. For details, please refer to Additional file 2.

#### Gene expression analysis

Raw gene expression data were normalized in batches using the RMA method [17], yielding normalized log2 intensities, and quality control (QC) reports were generated using the affyQCReport R package. Clustering analysis was performed as previously reported [18]. To identify differentially expressed genes, we used the Bayes moderate T-test implemented in the limma R package. Gene sets analysis using KEGG and Biocarta pathways as well as Gene Ontology terms, Molecular Signature Database gene sets and Stanford Microarray Database gene sets was performed on the 1000 genes most differentially expressed (500 genes up-regulated in responders and 500 genes up-regulated in non-responders) using hypergeometric tests. Custom gene sets were also built using Murat et al.'s data and included in the gene sets analysis [12]. In order to classify our samples according to Phillips et al.'s system, we used their expression data to build a centroid-based classifier, and after checking that it was able to reclassify Phillips et al.'s samples properly, we classified our series based on these centroids. In order to classify our samples according to Verhaak et al.'s system, we used their published centroid-based classifier [8,10].

#### CGHarray analysis

For CGHarray data, after a QC and filtering step, remaining spots were normalized using the lowess print-tip method, then replicated spots were averaged, yielding normalized log2 ratios. Smoothed log2 ratios were then obtained using the tilingArray R package. Gain and loss status, respectively, were determined for each clone based on a smoothed log2 ratio above (below) the distribution mode plus (minus) one standard deviation. Homozygous deletions and amplicons, respectively, were detected using a sample-based estimate of the tumor cell rate R_TC _as clones yielding smoothed log2 ratios below log2(1-R_TC_) (above log2(1+ 1.5* R_TC_)). Recurrent minimal genomic alterations (MCR) were obtained as previously described [19] . To identify clones or regions with differential genomic status, we used Fisher's exact test.

In all cases, control for multiple testing was done with the Benjamini and Hochberg approach.

Survival curves were calculated according to the Kaplan-Meier method, and differences between curves were assessed using the log-rank test.

### Independent data set

The influence of the *p16 *homozygous genomic deletions was studied in an independent data set of 222 GBMs from the Pitié-Salpêtrière Hospital's neuro-oncology department; the *p16 *deletion and MGMTP methylation statuses of these data set samples were available. This data set consists largely of patients whose data have been previously published [20]. These patients were treated with either radiation therapy alone or with radiation therapy followed by adjuvant chemotherapy with alkylating agents (BCNU or temozolomide).

## Results

### Classical and mesenchymal GBMs respond differently to radiotherapy and to first-line chemotherapy

**Figure 1:**
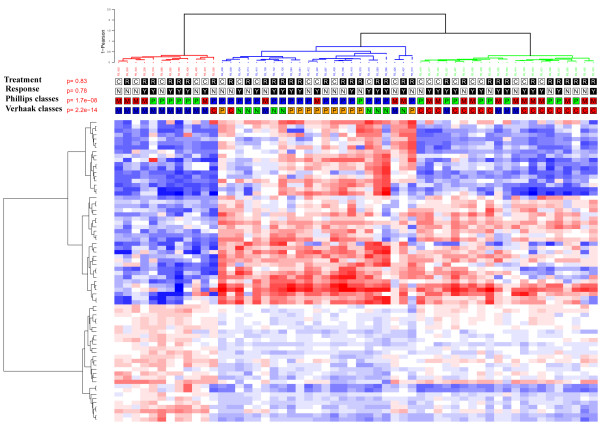
**Unsupervised clustering of the 56 glioblastomas**. Unsupervised hierarchical clustering of the 56 GBMs. The heatmap was done with the 72 probe sets used in the centroid classifier that was able to generate the 3 groups identified through unsupervised clustering (Additional file 1 Table S2). Samples and genes were clustered using Ward's linkage and 1-Pearson correlation coefficient. For each probe set, the lowest and highest intensity values are displayed in blue and red, respectively. Treatment: C = First-line chemotherapy, R = Radiotherapy. Response: N = non-responder, Y = responder. Phillips = class according to Phillips et al.'s classification [8], blue P = Proneural, green P = Proliferative, M = Mesenchymal. Verhaak = class according to Verhaak et al.'s classification [10], N = Neural, C = Classical, M = Mesenchymal, P = Proneural. The GBMs were classified into three groups: one group enriched in *EGFR*-amplified patients (n = 19, blue cluster), one group characterized by a high level of expression of immune and extra-cellular matrix genes (n = 12, red cluster) and one group characterized by a higher of expression of neural genes (n = 25, green cluster). There was a significant overlap when using Phillips et al.'s classes (Fisher's exact test p-value = 1.7 × 10^-8^) and a larger overlap with Verhaak et al.'s classes (Fisher's exact test p-value = 2.2 × 10^-14^).

First, in order to assess if responders and non-responders to either radiation therapy or first-line chemotherapy corresponded to different transcriptomic subgroups of GBMs, we performed an unsupervised hierarchical clustering analysis of the 56 GBMs. As shown in Figure 1, three main transcriptomic subgroups were identified. This clustering was robust and conserved across different gene lists and clustering methods. The centroids generating this classification are provided in Additional file 1 Table S2. However, none of the three clusters was enriched in responders or non-responders, and neither the PFS nor the OS differed between the three clusters. As shown in Figure 1, some responders and non-responders had very similar gene expression profiles.

**Figure 2:**
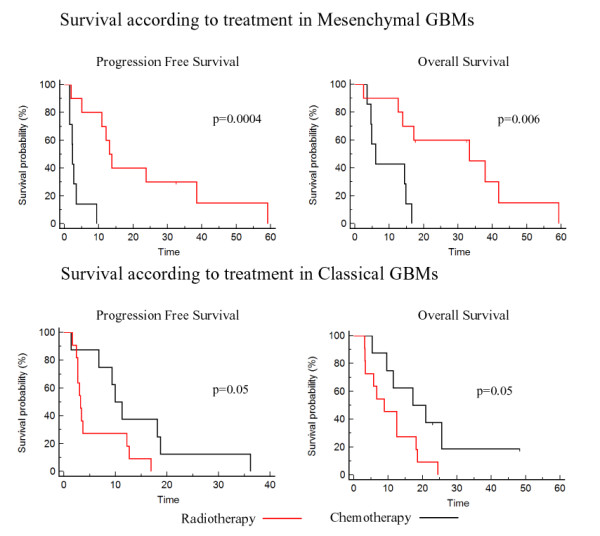
**Survival according to treatment in mesenchymal and classical GBMs**. Progression-free survival and overall survival according to treatment (radiotherapy = red, first-line chemotherapy = black) in the GBMs of the present study classified as mesenchymal or classical according to Verhaak et al.'s classes [10].

Next, in order to assess if transcriptomic subgroups of GBMs previously identified in larger series of patients were associated with a specific pattern of response to radiotherapy or to chemotherapy, we classified our 56 samples according to Phillips et al.'s and Verhaak et al.'s transcriptomic classifications and estimated the response rate in each subgroup [8,10]. As shown in Figure 1, there was a significant but not complete overlap between our three subgroups of GBMs and the classes of GBMs identified in these studies. As shown in Table 1, Verhaak et al.'s classes (but not Phillips et al.'s classes) were significantly associated with response to treatment. Indeed, we found that GBMs classified as mesenchymal were more likely to respond to radiotherapy (8 out of 10) than to chemotherapy (1 out of 7) (Fisher's exact test, p = 0.01), whereas GBMs classified as classical were more likely to respond to chemotherapy (7 out of 8) than to radiotherapy (3 out of 11) (Fisher's exact test, p = 0.02). Accordingly, as shown in Figure 2, patients with a mesenchymal GBM had a better outcome when treated with radiotherapy, whereas patients with a classical GBM had a better outcome when treated with first-line chemotherapy. In the neural GBMs, the overall response rate to either radiotherapy or chemotherapy was higher than in the other subgroups (8 responders out of 9 neural GBMs versus 23 responders out of 47 non-neural GBMs, Fisher's exact test, p = 0.03).

**Table 1:** Response to radiotherapy according to Phillips and Verhaak classifications

	Response to chemotherapy		Response to radiotherapy		Fisher's exact test
	No	Yes	No	Yes	

Phillips subgroups					
Mes.	4	7	5	6	NS
PN	2	3	6	7	NS
Proliferative	4	2	4	6	NS
					
Verhaak subgroups					
Mes.	**6**	1	2	**8**	p = 0.01*
Neural	0	**3**	1	**5**	p = 0.03^°^
PN	3	1	4	3	NS
Classical	1	**7**	**8**	3	P = 0.02*

Mes. = Mesenchymal, PN = Proneural

* comparison of the frequencies of response to chemotherapy and to radiotherapy in mesenchymal and classical GBMs

° comparison of the frequency of response to treatment in neural vs. other classes of GBMs

### Molecular characteristics associated with response to radiation therapy

**Figure 3:**
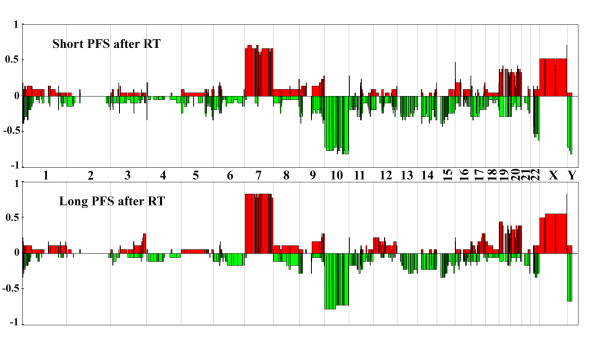
**Genomic profiles of patients with short and long PFS after radiation therapy**. CGH array genomic profiles of the patients with short (< 5 months) and long (> 10 months) PFS after radiotherapy. For each chromosome, the telomere of the short arm is on the left and the telomere of the long arm is on the right. Genomic gains and losses are shown in red and green, respectively. The y-axis corresponds to the frequency of gains and losses in each group of patients.

In a second step, we focused on all patients treated with radiotherapy alone to identify the molecular characteristics associated with a response to radiation therapy. Comparison of the genomic profiles of responders (n = 21) and non-responders (n = 18) demonstrated that the two groups of patients had very similar genomic profiles (Figure 3). In both groups, there was a high frequency of chromosome 7 gain and chromosome 10 loss, consistent with the most frequently observed genomic abnormalities in GBMs. Only three Minimal Common Regions (MCR) were significantly different between the two groups (Fisher's exact test p-value <0.05), albeit at a low frequency (Table 2). Several genes located in these MCRs were also differentially expressed, but to our knowledge, none of them has been reported to play a role in response to radiation therapy (Table 2 and Additional file 1 Table S3).

**Table 2:** Minimal Common Regions differentiating responders from non-responders to radiation therapy and first-line chemotherapy

Event	Chr	Treatment			Differentially expressed genes located in the MCR
		**Radiation therapy**			
		**% in NR**	**% in R**	**p-value**	

Loss	5p15.3	0	33	0.04	

Loss	5p15.2-3	0	29	0.01	CMBL (FC = 1.3)

Gain	17q24-25	22	0	0.02	TANC2 (FC = 1.1), DCAF7 (FC = 1.2), LRRC37A (FC = 1.3), PITPNC1 (FC = 1.6)

					

		**Chemotherapy**			
		**% in NR**	**% in R**	**p-value**	

Amp.	7p11.2	0.35	0.81	0.02	

Del.	9p21.3	0	0.82	< 10^-4^	MTAP (FC = 2), CDKN2A (FC = 2.6)

Loss	9p24	0.12	0.64	0.01	

Loss	9p24	0.12	0.64	0.01	KIAA1432 (FC = 1.4), KDM4C (FC = 1.2)

Loss	9p24;p23;p22	0.12	0.64	0.01	ZDHHC21 (FC = 1.6)

Gain	11p15	0.18	0.64	0.02	PGAP2 (FC = 0.8)

Gain	11q13	0.18	0.91	0.0003	C11orf68 (FC = 0.8), B3GNT1 (FC = 0.75), RAB1B (FC = 0.86)

Gain	17p13	0.18	0.64	0.02	CRK (FC = 0.83), INPP5K (FC = 0.88), TSR1 (FC = 0.87), METT10 D (FC = 0.77), SGSM2(FC = 0.75)

Gain	17p13	0.18	0.64	0.02	METT10 D (FC = 0.77)

Gain	19p13.3	0.35	0.91	0.006	PPAP2C (FC = 0.74), SHC2 (FC = 0.8), MOBKL2A (FC = 0.81), SCAMP4 (FC = 0.8), BTBD2 (FC = 0.8), FAM108A1 (0.86), SF3A2 (FC = 0.74), AP3D1 (0.77), GNG7 (0.46), ZNF555 (0.82), DOHH (0.82), C19orf29 (0.8), NFIC (0.75)

Gain	19p13.3;p13.2;p13.1	0.29	0.91	0.002	SLC25A23 (0.6), CLEC4 M (0.85), ZNF846 (0.7), OLFM2 (0.6), S1PR2 (0.8), DHPS (0.85), TNPO2 (0.76), ZNF791 (0.77)

Gain	19p13.1	0.29	0.91	0.002	EPS15L1 (0.83), OCEL1 (0.85), TMEM161A (0.82), NCAN (0.3)

Gain	19p12	0.29	0.91	0.002	

Gain	19p12;p11	0.29	0.91	0.002	

Gain	19q11;q12	0.29	0.82	0.002	FXYD3 (0.84)

Gain	19q12; q13.1	0.35	0.91	0.006	FAM98C (0.77)

Gain	20p13	0.12	0.82	0.0004	ZCCHC3 (0.81), SOX12 (0.84), RBCK1 (0.8), CSNK2A1 (0.81), MAVS (0.8), ATRN (0.76)

Gain	20p12.3; p11.2	0.18	0.64	0.002	RRBP1 (0.73)

Gain	20p11.2	0.12	0.64	0.003	

Gain	20p11.2; p11.1; q11.1;q11.2	0.24	0.73	0.02	CST8 (0.87)

Gain	20q11.2	0.24	0.82	0.006	PLUNC (0.88), CHMP4B (0.83)

Gain	20q11.2	0.24	0.82	0.006	CHMP4B (0.83), LOC647979 (0.67)

Gain	20q11.2;q12	0.18	0.73	0.006	SNHG11 (0.75)

Gain	20q13.1	0.18	0.73	0.006	SLC13A3 (0.81)

Gain	20q13.1	0.24	0.73	0.02	PREX1 (0.72)

Gain	20q13.3	0.24	0.73	0.02	CDH4 (0.5), OSBPL2 (0.82)

Chr= chromosome, NR= Non-responder, R= Responder, % = Frequency of the event, FC= Fold change in NR versus R, Amp. = Amplification, Del. = Homozygous deletion

Recurrent alterations were defined for the entire population of samples if the identical alteration was present in at least two samples. Computation of recurrent minimal genomic alterations was done in a similar way to a method previously described using original R code [19]. Comparison of the genomic profiles of responders (n = 21) and non-responders (n = 18) demonstrated that three Minimal Common Regions (MCRs) were significantly different between the two groups (Fisher's exact test p-value <0.05). Comparison of the genomic profiles of responders (n = 11) versus non-responders (n = 17) to first-line chemotherapy demonstrated substantial genomic differences, with twenty-four MCRs being significantly (p < 0.05) associated with the chemotherapy response. Differentially expressed genes with a p-value <0.05 and located in the MCR are shown with their corresponding fold changes.

Therefore, we focused on the comparison of the gene expression profiles of responders (n = 19) and non-responders (n = 15). As suggested by the observation that responders and non-responders to radiotherapy could have very similar expression profiles, the differences between responders and non-responders were modest; nevertheless, 417 genes were up-regulated in non-responders and 449 up-regulated in responders with p < 0.05 and fold change > 1.5 (Additional file 1 Table S4). To characterize the differences between the two groups, we performed a gene set analysis on the 1000 genes most differentially expressed (500 genes up-regulated in responders and 500 genes up-regulated in non-responders). This demonstrated that these gene lists were enriched in genes with very different ontologies (Table 3, Table 4 and Additional file 1 Table S5). The list of up-regulated genes in responders was most significantly enriched in genes involved in the immune response, namely in immune genes previously reported to be associated with an improved outcome after radiochemotherapy (Cluster G24, Table 4) [12]. This enrichment was seen in non-specific inflammatory response genes as well as in genes involved in the B cell-mediated response and T cell activation (Table 3, Table 4). In order to validate these findings at the protein level, an immunohistochemical study of CD3, CD20 and CD68 markers was performed in the tumors of responders and non-responders to radiotherapy. Neither GBM samples of responders nor those of non-responders were infiltrated by CD20 cells. However, infiltration by CD3 cells and by both CD3 and CD68 was much more frequent in responders than in non-responders to radiotherapy (Table 5, Fisher's exact test, p-value = 0.04). On the other hand, the list of up-regulated genes in non-responders was most significantly enriched in genes induced by hypoxia, suggesting a higher level of hypoxia in the non-responders (Table 3, Table 4). As hypoxia is a well-known mechanism of radiation resistance, these results suggest that even in GBMs, which are highly hypoxic tumors, a higher level of hypoxia raises the level of resistance to radiation therapy.

**Table 3:** Summary of the most relevant gene sets enriched in responders and non-responders to radiotherapy

Gene sets most significantly enriched in responders	BH adjusted p-value	Gene sets most significantly enriched in non-responders	BH adjusted p-value
Murat et al. immune gene cluster (G24)	< 10^-4^	MSigDB C2 pathways MENSE_HYPOXIA_UP	< 10^-4^
GO:0042613 - MHC class II protein complex	< 10^-4^	SMD processes core_hypoxia1_sw	< 10^-4^
SMD cancerModules Immune (humoral) and inflammatory response	< 10^-4^	MSigDB C2 pathways HYPOXIA_REVIEW	0.001
GO:0006955 - immune response	< 10^-4^	SMD cancerModules DRG (dorsal root ganglia) genes	0.006
MSigDB C2 pathways LEE_TCELLS2_UP	< 10^-4^	MSigDB C2 pathways HYPOXIA_REG_UP	0.01
GO:0006954 - inflammatory response	< 10^-4^		
GO:0045087 - innate immune response	< 10^-4^		

Summary of gene sets analysis using KEGG, Biocarta pathways, Gene Ontology (GO) terms, Molecular Signatures Database (MSigDB) gene sets, Stanford microarray database (SMS) and Murat et al.'s gene sets [12]. BH: Benjamini and Hochberg.

**Table 4:** Most differentially expressed genes between responders and non-responders to radiotherapy

Twenty most up-regulated genes in responders to radiotherapy				
**Probe set**	**Gene symbol**	**Description**	**FC**	**p-value**

211538_s_at	HSPA2	Heat shock 70 kDa protein 2	3.4	< 10^-4^
209687_at	CXCL12	Chemokine ligand 12 (SDF1)	2.6	0.001
209480_at	HLA-DQB1	MHC complex, class II, DQ beta 1	2.5	0.023
221900_at	COL8A2	Collagen, type VIII, alpha 2	2.5	0.004
226818_at	MPEG1	Macrophage expressed gene 1	2.4	< 10^-4^
219759_at	ERAP2	Endoplasmic reticulum aminopeptidase 2	2.3	0.019
220146_at	TLR7	Toll-like receptor 7	2.3	0.002
222881_at	HPSE	Heparanase	2.2	< 10^-4^
239270_at	PLCXD3	Phosphatidylinositol-specific Phospholipase C, × domain containing 3	2.2	0.001
218858_at	DEPDC6	DEP domain containing 6	2.2	0.001
209343_at	EFHD1	EF-hand domain family, D1	2.2	0.004
205034_at	CCNE2	Cyclin E2	2.2	0.004
223170_at	TMEM98	Transmembrane protein 98	2.2	0.012
203184_at	FBN2	Fibrillin 2	2.2	0.020
1557395_at	LOC255130		2.2	0.035
204466_s_at	SNCA	Synuclein, alpha	2.1	0.017
228598_at	DPP10	Dipeptidyl-peptidase 10	2.1	0.023
1567628_at	CD74	CD74 molecule, MHC complex	2.1	0.006
219750_at	TMEM144	Transmembrane protein 144	2.1	0.04
228376_at	GGTA1	Glycoprotein, alpha-galactosyltransferase 1	2.1	0.006

**Twenty genes most differentially up-regulated in non-responders to radiotherapy**				

220405_at	SNTG1	Syntrophin, gamma 1	3.4	0.001
236761_at	LHFPL3	Lipoma HMGIC fusion partner-like 3	3.2	0.035
204913_s_at	SOX11	SRY (sex determining region Y)-box 11	3.1	0.038
205230_at	RPH3A	Rabphilin 3A homolog (mouse)	2.8	0.014
202859_x_at	IL8	Interleukin 8	2.8	0.023
206201_s_at	MEOX2	Mesenchyme homeobox 2	2.7	0.047
230498_at	MCHR1	Melanin-concentrating hormone receptor 1	2.7	0.006
1554452_at	HIG2	Hypoxia-inducible protein 2	2.7	0.003
206984_s_at	RIT2	Ras-like without CAAX 2	2.6	0.023
225285_at	BCAT1	Branched chain aminotransferase 1, cytosolic	2.4	< 10^-4^
223278_at	GJB2	Gap junction protein, beta 2, 26kDa	2.4	0.021
205358_at	GRIA2	Glutamate receptor, ionotropic, AMPA 2	2.4	0.027
227361_at	HS3ST3B1	Heparan sulfate (glucosamine) 3-O-sulfotransferase 3B1	2.4	0.038
211527_x_at	VEGFA	Vascular endothelial growth factor A	2.4	0.015
219196_at	SCG3	Secretogranin III	2.3	0.025
232099_at	PCDHB16	Protocadherin beta 16	2.3	0.010
217562_at	FAM5C	Family with sequence similarity 5, member C	2.3	0.023
214920_at	THSD7A	Thrombospondin, type I, domain containing 7A	2.3	0.025
202499_s_at	SLC2A3	Solute carrier family 2 (facilitated glucose transporter), member 3	2.2	< 10^-4^
202912_at	ADM	Adrenomedullin	2.2	0.016

List of the 20 genes most up-regulated and with a p-value <0.05 in responders versus non-responders and in non-responders versus responders to radiotherapy. FC: Fold change, p-value: Limma T-test p-value. Immune genes and hypoxia genes are underlined in responders and non-responders, respectively.

**Table 5:** CD20, CD3 and CD68 immunohistochemistry in responders and non-responders to radiation therapy

	Responders			Non-responders			Fisher's exact test
	**Negative**	**+**	**++**	**Negative**	**+**	**++**	**p-value**

**CD20**	15	0	0	10	0	0	NS
**CD3**	4	6	5	7	3	0	0.04
**CD68**	5	7	3	6	4	1	NS
**CD3 and CD68**	5	10		8	2		0.04

### Molecular characteristics associated with response to first-line chemotherapy

**Figure 4:**
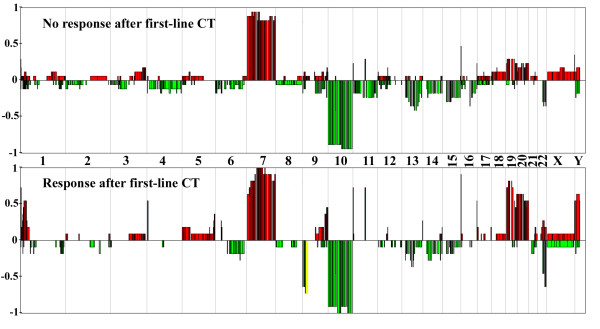
**Genomic profiles of non-responders and responders to first-line chemotherapy**. CGH array genomic profiles of the non-responders and the responders to first line chemotherapy. For each chromosome, the telomere of the short arm is on the left and the telomere of the long arm is on the right. Genomic gains and losses are shown in red and green, respectively. *p16 *locus homozygous deletion is shown in yellow. The y-axis corresponds to the frequency of gains and losses in each group of patients.

Comparison of the genomic profiles (gains, losses, homozygous deletions and amplifications) of responders (n = 11) versus non-responders (n = 17) to first-line chemotherapy (in contrast to radiation therapy) demonstrated substantial genomic differences (Figure 4). *CDKN2A (p16) *locus homozygous deletions on 9p21, *EGFR *amplification and 24 MCRs (3 loss and 21 gains) were significantly associated with response to chemotherapy (Fisher's exact test, p < 0.05) (Table 2 and Additional file 1 Table S6). *CDKN2A *(*p16*) locus homozygous deletion was the most significant event; it was observed in 82% of responders but in none of the non-responders (Fisher's exact test, p < 10^-4^). It was associated with a significant down-regulation of the expression of CDKN2A in responders (p = 0.04). Concerning *MTAP *and *CDKN2B*, which are also located in the *p16 *locus, a significant down-regulation of the expression in responders was observed only for MTAP (p = 0.0003). Among the other genomic alterations that have been reported to alter the retinoblastoma (RB) signaling pathway in GBMs (i.e*., CDK4 *or *CDK6 *or *CCND2 *amplification and *RB1 *or *CDKN2C *homozygous deletion), we found a *CDK4 *amplification in three non-responders and a *CCND2 *amplification in one responder who had also a *p16 *deletion. Thus, in contrast to *p16 *deletion, other genomic alterations disrupting the RB pathway did not seem to be associated with response to chemotherapy.

*EGFR *amplification was more frequently observed in responders than in non-responders (81% vs. 35%, Fisher's exact test, p = 0.02). All lost MCRs were located on chromosome 9p22-24, and these loci were not independent. Among the genes located in these MCRs (Table 2), none has been reported to be involved in chemosensitivity. Gained MCRs were located on chromosomes 11p, 11q, 17p, 19p, 19q, 20p and 20q. Genes located in these MCRs and significantly overexpressed in responders are summarized in Table 2. To our knowledge, none of these genes has been associated with chemosensitivity.

Surprisingly, MGMTP methylation analysis demonstrated that most patients in this series were MGMTP unmethylated (8 out of 11 responders and 14 out of 17 non-responders); thus, these genomic abnormalities may actually represent alternative mechanisms of chemosensitivity in MGMTP unmethylated patients.

Next, we compared the gene expression profiles of responders (n = 12) and non-responders (n = 10). The differences between the two groups were less important than the analogous differences between responders and non-responders to radiotherapy, with 292 genes being up-regulated in non-responders and 203 genes being up-regulated in responders with a p-value <0.05 and a fold change >1.5 (Additional file 1 Table S7). Gene set analysis on the 1000 genes most differentially expressed between the two groups (500 genes up-regulated in responders and 500 genes up-regulated in non-responders) was performed. In agreement with the genomic analysis, the list of up-regulated genes in non-responders was enriched in genes located on 9p (Table 6). Interestingly, this gene list was also enriched in genes reported to be up-regulated in embryonic and neural stem cells, whereas the list of genes up-regulated in responders was enriched in genes up-regulated in the normal brain, suggesting a link between resistance to chemotherapy and a more undifferentiated phenotype of the tumor (Table 6, Table 7 and Additional file 1 Table S8). Consistently, the list of genes up-regulated in responders was enriched in a set of normal brain genes (Cluster 18, Table 7) associated with improved outcome after chemoradioherapy whereas the list of genes up-regulated in non-responders was enriched in a set of stem-cell genes (Cluster 28_98, Table 7) associated with a worse outcome after concomitant chemoradiotherapy [12]. Among the stem-cell genes, HOXA10 and HOXC6, were the most up-regulated genes in non-responders to chemotherapy.

**Table 6:** Summary of the most relevant gene sets enriched in responders and non-responders to chemotherapy

Gene sets most significantly enriched in responders	BH adjusted p-value	Gene sets most significantly enriched in non-responders	BH adjusted p-value
MSigDB C2 pathways AGEING_BRAIN_UP	< 10^-4^	SMD chromArms 9p	0.005
SMD cancerModules CNS genes	< 10^-4^	MSigDB C2 pathways STEMCELL_NEURAL_UP	0.007
Murat et al. normal brain gene cluster (G18)	< 10^-4^	MSigDB C2 pathways LEE_TCELLS2_UP	0.01
SMD tissues Brain_sw	0.06	GO:0001952 - regulation of cell-matrix adhesion	0.02
		MSigDB C2 pathways STEMCELL_EMBRYONIC_UP Murat et al. stem cell gene cluster (G28_G98)	0.02 1

Summary of gene sets analysis using KEGG, Biocarta pathways, Gene Ontology (GO) terms, Molecular Signatures Database (MSigDB) gene sets and Stanford microarray database (SMD) and Murat et al.'s gene sets [12]. BH: Benjamini and Hochberg.

**Table 7:** Most differentially expressed genes between responders and non-responders to first-line chemotherapy

Twenty genes most up-regulated in responders to chemotherapy				
**Probe set**	**Gene symbol**	**Description**	**FC**	**p-value**

203296_s_at	ATP1A2	ATPase, Na+/K+ transporting, alpha 2 (+) polypeptide	5.4	0.003
230865_at	LIX1	Lix1 homolog (mouse)	3.8	0.005
209728_at	HLA-DRB4	MHC, DR beta 4	3.8	0.002
223075_s_at	AIF1L	Allograft inflammatory factor 1-like	3.6	0.002
210738_s_at	SLC4A4	Solute carrier family 4, sodium bicarbonate cotransporter, member 4	3.5	0.02
223434_at	GBP3	Guanylate binding protein 3	3.5	0.01
228581_at	KCNJ10	Potassium inwardly-rectifying channel, subfamily J, member 10	3.4	0.008
229778_at	C12orf39	chromosome 12 open reading frame 39	3.3	0.02
205143_at	NCAN	Neurocan	3.3	0.02
225911_at	NPNT	Nephronectin	3.3	0.004
209074_s_at	FAM107A	Family with sequence similarity 107, member A	3.3	0.004
206306_at	RYR3	Ryanodine receptor 3	3.1	0.04
217057_s_at	GNAS	GNAS complex locus	3.1	0.03
220029_at	ELOVL2	Elongation of very long chain fatty acids (FEN1/Elo2, SUR4/Elo3, yeast)-like 2	3.1	0.008
1558010_s_at	SLC1A2	Solute carrier family 1 (glial high affinity glutamate transporter), member 2	3.1	0.01
223699_at	CNDP1	Carnosine dipeptidase 1 (metallopeptidase M20 family)	2.9	0.02
211597_s_at	HOPX	Homeodomain-only protein	2.8	0.002
209631_s_at	GPR37	G protein-coupled receptor 37 (endothelin receptor type B-like)	2.8	0.002
204379_s_at	FGFR3	Fibroblast growth factor receptor 3	2.8	0.03
214279_s_at	NDRG2	NDRG family member 2	2.7	0.008

**Twenty genes most up-regulated in non-responders to chemotherapy**				

210809_s_at	POSTN	Periostin, osteoblast specific factor	4.3	0.05
223278_at	GJB2	Gap junction protein, beta 2, 26kDa	4.1	0.01
231735_s_at	MALAT1	Metastasis associated lung adenocarcinoma transcript 1	3.6	0.003
206785_s_at	KLRC1///KLRC2	Killer cell lectin-like receptor subfamily C, member 1///member 2	3.3	0.03
230472_at	IRX1	Iroquois homeobox protein 1	3.1	0.03
213350_at	RPS11	Ribosomal protein S11	2.8	0.004
228367_at	ALPK2	Alpha-kinase 2	2.7	0.01
209644_x_at	CDKN2A	Cyclin-dependent kinase inhibitor 2A (p16)	2.7	0.04
221872_at	RARRES1	Retinoic acid receptor responder (tazarotene induced) 1	2.6	0.04
224321_at	TMEFF2	Transmembrane protein with EGF-like and two follistatin-like domains 2	2.5	0.004
225314_at	OCIAD2	OCIA domain containing 2	2.5	0.02
206858_s_at	HOXC6	Homeobox C6	2.5	0.01
213150_at	HOXA10	Homeobox A10	2.4	0.04
235412_at	ARHGEF7	Rho guanine nucleotide exchange factor (GEF) 7	2.4	0.03
209687_at	CXCL12	Chemokine (C-X-C motif) ligand 12	2.3	0.03
206282_at	NEUROD1	Neurogenic differentiation 1	2.2	0.02
227388_at	TUSC1	Tumor suppressor candidate 1	2.2	0.0007
1562403_a_at	SLC8A3	Solute carrier family 8 (sodium-calcium exchanger), member 3	2.2	0.02
201387_s_at	UCHL1	Ubiquitin carboxyl-terminal esterase L1 (ubiquitin thiolesterase)	2.2	0.01
231984_at	MTAP	Methylthioadenosine phosphorylase	2.0	0.0003

List of the 20 genes most up-regulated and with a p-value <0.05 in responders versus non-responders and in non-responders versus responders to first-line chemotherapy. FC: Fold change, p-value: Limma T-test p-value. Normal brain genes and stem-cell genes are underlined in responders and non-responders, respectively.

### Impact of *p16 *locus homozygous deletions on outcome after adjuvant chemotherapy in an independent data set

As the *p16 *locus homozygous deletion was the most consistent finding in patients responding to first-line chemotherapy, we focused on the study of this genomic abnormality in an independent series of patients treated either by radiotherapy alone (n = 79) or by radiotherapy followed by adjuvant chemotherapy (n = 143) for whom the MGMTP methylation status was also available (Table 8). We hypothesized that, if this genomic abnormality was consistently associated with chemosensitivity, then, as has been demonstrated for MGMTP methylation, the benefit of adjuvant chemotherapy would be more pronounced in the group of patients with the *p16 *deletion.

**Table 8:** Characteristics of the patients from the Pitié-Salpêtrière database

Treatment group		Age (median)	Karnofsky (median)	Surgery (% B/P/C)	MGMTP methylated
P16 not deleted (n = 141)					
RT alone	N = 47	57	80	8/27/65	55%
RT+CT	N = 94	55	80	13/24/63	46%
P16 deleted (n = 81)					
RT alone	N = 32	57	80	15/25/60	53%
RT+CT	N = 49	57	80	10/27/63	53%

Surgery (B/P/C): Percentage of Biopsy/Partial/Complete surgery; MGMTP methylated: percentage of patients with methylated MGMT; RT: radiotherapy; RT+CT: radiotherapy and adjuvant chemotherapy.

**Figure 5:**
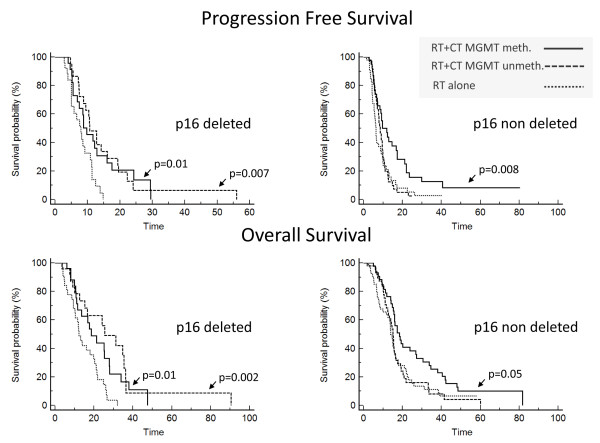
**Survival according to treatment, radiotherapy alone or radiotherapy and adjuvant chemotherapy, *p16 *deletion and MGMTP methylation in an independent series of 222 GBMs**. The y-axis corresponds to the survival probability and the x-axis to survival time (months). Survival curves on the left correspond to the patients with *p16 *deletions; survival curves on the right correspond to patients without *p16 *deletions. These survival curves show that patients with *p16 *deletions benefit from adjuvant chemotherapy regardless of their MGMTP methylation status, whereas patients without *p16 *deletions benefit from adjuvant chemotherapy only when they have a methylated MGMTP.

In the patients with the *p16 *deletion, we found that, regardless of MGMTP methylation status, those treated with radiotherapy and adjuvant chemotherapy had a significantly longer PFS (10.7 vs. 7.9 months, p = 0.007, if unmethylated; 9.7 vs. 7.9 months, p = 0.01, if methylated) and OS (25.3 vs. 12.2 months, p = 0.002, if unmethylated; 18.6 vs. 12.2 months, p = 0.01, if methylated) than those treated with radiotherapy alone (Figure 5). This association was independent of age, Karnofsky performance status and type of surgery. In contrast, among patients without the *p16 *deletion who were treated with radiotherapy and adjuvant chemotherapy, only those patients with a methylated MGMTP had a longer PFS (11.8 vs. 6.3 months, p = 0.008) and a longer OS (18.4 vs. 15 months, p = 0.05) than those treated with radiotherapy alone (Figure 5). Patients without the *p16 *deletion treated with radiotherapy and adjuvant chemotherapy and with an unmethylated MGMTP did not fare better than patients without the *p16 *deletion who were treated with radiotherapy alone.

The finding that MGMTP unmethylated patients benefit from adjuvant chemotherapy when the *p16 *locus is deleted is consistent with the association between *p16 *deletion and chemosensitivity. However, we found no additive effect between MGMTP methylation and *p16 *deletion. Indeed, in the group of patients with a *p16 *deletion who were treated with radiotherapy and adjuvant chemotherapy, the outcomes were similar between MGMTP methylated and unmethylated patients (Figure 5).

## Discussion

Several microarray studies have focused on the relationship between gene expression profiles, genomic profiles and overall survival in high-grade gliomas [4,6-10,21], but few studies have used microarray technology to describe the molecular characteristics associated with the response of the tumor to therapy [11,12]. A recent study examined the gene expression profile and survival in GBM patients treated with either radiation therapy alone or with concomitant chemoradiotherapy with temozolomide, but this study essentially focused on patients treated with concomitant chemoradiotherapy [12]. Thus, until now, no study had performed an extensive microarray-based study of the gene expression and genomic characteristics of GBMs with different responses after either radiation therapy alone or first-line chemotherapy. The present study has several limitations. Beside the limited number of patients, the response criteria used to define response to radiotherapy are debatable. Furthermore, the fact that most responders to first-line chemotherapy were MGMTP unmethylated suggests that this group of patients might not be completely representative of the entire population of GBMs. However, despite these limitations, the present study provides new information regarding the molecular characteristics associated with responses to radiotherapy and to first-line chemotherapy in GBMs.

Our first objective was to assess if responders and non-responders to radiotherapy or to chemotherapy corresponded to distinct transcriptomic subgroups of GBMs. Using unsupervised hierarchical clustering, we were unable to identify any transcriptomic subgroups of responders or non-responders. However, we found an association between Verhaak et al.'s transcriptomic classification of GBMs and the response rates to radiotherapy and to chemotherapy [10]. This classification has been established by the Cancer Genome Atlas Network and is presently the most accomplished transcriptomic classification system for GBMs [10]. It distinguishes four subgroups of GBMs. Classical GBMs are characterized by a 95% rate of *EGFR *amplification, *p16 *locus deletion and an astrocytic gene expression profile. Mesenchymal GBMs are characterized by a high rate of *NF1 *mutation/deletion and an activated astroglial gene expression profile. Proneural GBMs are characterized by a high rate of *PDGFRA *amplification and *IDH1 *mutations and an oligodendroglial gene expression profile. Neural GBMs are characterized by a normal brain-like gene expression profile [10]. Using this classification, it has been suggested that classical and mesenchymal GBMs, unlike proneural GBMs, benefit from more aggressive treatment consisting of concomitant chemoradiotherapy or radiotherapy followed by prolonged adjuvant chemotherapy (> 3 cycles). However, there are currently no data concerning the response rates of these subgroups of GBMs to either radiotherapy alone or to first-line chemotherapy. Interestingly, we found that classical GBMs were more likely to respond to first-line chemotherapy than to radiotherapy (87.5% vs. 27%, p = 0.02), whereas mesenchymal GBMs were more likely to respond to radiotherapy than to first-line chemotherapy (80% vs. 15%, p = 0.01). We also observed that neural GBMs had higher response rates to both radiotherapy and chemotherapy than the other subtypes (88% versus 49%, p = 0.03). Of course, these results must be considered with caution because of the limited number of GBMs studied and because of the selection criteria used in the present study. However, in line with Verhaak et al.'s findings, these results suggest that these transcriptomic subgroups of GBMs might benefit from different therapeutic strategies.

In our cohort, the transcriptomic and genomic characteristics associated with a therapeutic response differed between patients treated with radiation therapy and patients treated with first-line chemotherapy. These results are in agreement with a recent study showing that the molecular characteristics predictive of resistance to chemoradiotherapy were not predictive of resistance to radiation therapy alone [12]. They are also in agreement with the finding that in the present study, responders to radiotherapy were mostly mesenchymal GBMs, whereas responders to chemotherapy were mostly classical GBMs, though this might also be related to the different criteria used to define response in our two groups of patients.

In examining the differences between responders and non-responders to radiation therapy, our main finding is relevant to the differential expression of genes implicated in the micro-environment. As observed in another study, genes implicated in the innate immune response were enriched in responders to radiation therapy [12]. Consistently, we found that the tumors of responders were much more frequently infiltrated by both T lymphocytes and microglial cells than were those of non-responders. This finding is also in agreement with the high response rate to radiotherapy observed among the mesenchymal GBMs. Indeed, these GBMs are characterized by a high level of inflammation and microglial infiltration [10]. This finding suggests that stimulating innate immunity might enhance tumor control and encourage the use of immunotherapy [22]. It is also interesting to link this result with epidemiological studies that have demonstrated a lower incidence of gliomas in patients with allergies, as these studies suggest that particular immune characteristics may protect against gliomas [23]. Recently, the expression of inflammation genes in gliomas has also been inversely correlated with the expression of CD133, a marker of stem cells [24]. On the other hand, hypoxia-induced genes were enriched in non-responders to radiation therapy. Hypoxia is a well known factor involved in radiation resistance [25]. GBMs are highly hypoxic tumors, and hypoxia plays a key role in their pathogenesis [26]. It enhances angiogenesis and selects for highly malignant cells resistant to hypoxic cell death and with increased migration capabilities [26]. Furthermore, hypoxia has been shown to maintain tumor stem cells in GBMs, which have been suggested to promote radioresistance [27]. Hypoxia gene expression profiles have been shown to be associated with poor outcome in several cancers, but not in GBMs [28]. The volume of the hypoxic tumor and the maximum level of hypoxia in GBMs measured using [18F]fluoromisonidazole positron emission tomography have been associated with a shorter PFS and OS after radiation therapy [29], and similar results have been obtained using binding of EF5 to measure tissue hypoxia [30]. However, up to now, therapeutic strategies aiming at reducing hypoxia to increase the efficacy of radiation therapy have failed to demonstrate efficacy in gliomas [31]. Interestingly, in our series, VEGFA was one of the most overexpressed hypoxia-induced genes in non-responders. VEGFA enhances endothelial cell survival, proliferation, migration and blood vessel permeability [32,33]. This in turn contributes to heterogeneous oxygen delivery and hypoxia [34]. Anti-VEGF treatments have been demonstrated to normalize the structure and function of the abnormal neovasculature [34-36], thus restoring normal blood flow, diminishing vascular leakage, reducing hypoxia and ultimately making tumor cells more sensitive to radiation therapy. Therefore, the overexpression of VEGFA and hypoxia-induced genes in non-responders to radiation therapy in our cohort supports the use of therapies that combine anti-angiogenics with radiation therapy to enhance the efficacy of radiotherapy by reducing tumor hypoxia [35].

In examining the differences between responders and non-responders to first-line chemotherapy, we found that the genes up-regulated in non-responders were enriched in neural and embryonic stem-cell genes, whereas those up-regulated in responders were enriched in normal brain genes. This is consistent with the finding in another study that a stem-cell gene expression profile was associated with shorter survival after combined radiochemotherapy [12] and with several reports suggesting that cancer stem cells are more resistant to chemotherapy [37-39].

Another interesting result of our study is that the most striking genomic difference between responders and non-responders to first-line chemotherapy was the overrepresentation of homozygous *p16 *locus deletions in responders. *P16/CDKN2A *deletions occur in about 30-50% of GBMs but in 95% of classical GBMs [10,40]. Consistently, we found that classical GBMs were more likely to respond to first-line chemotherapy. The association between *p16 *deletions and enhanced chemosensitivity to adjuvant temozolomide has already been suggested [41]. Several experimental studies have also suggested that p16 expression is associated with chemoresistance in gliomas [42-44]. In order to validate the association between *p16 *deletion and chemosensitivity, we assessed the impact of *p16 *deletion in an independent series of patients treated either with radiation therapy alone or with radiation therapy and adjuvant chemotherapy. In this dataset, we observed a benefit of adjuvant chemotherapy in both MGMTP methylated and unmethylated patients when *p16 *was deleted, whereas this benefit was only observed in MGMTP methylated patients when *p16 *was not deleted, supporting the association between *p16 *deletion and chemosensitivity. This also suggests that *p16 *deletion might be an alternative mechanism of chemosensitivity in MGMTP unmethylated patients and is consistent with our finding that most of the responders to first-line chemotherapy were MGMTP unmethylated.

The present study demonstrates that the response of GBMs to either first-line chemotherapy or radiotherapy relies on different molecular mechanisms. According to their transcriptomic and genomic characteristics, some patients benefit more from chemotherapy and others more from radiotherapy. These results encourage the combination of both treatment strategies to increase the likelihood of treatment response and suggest that prognostic markers could be identified both to predict chemo-or radiotherapy efficacy and to develop new therapeutic strategies.

## Competing interests

The authors declare that they have no competing interests.

## Authors' contributions

FD and AdR performed the majority of the experiments and analysis. FD, AdR, JH, MS and FB drafted the manuscript. DFB and CC performed the immunohistochemistry study. AI and YM performed the analysis of the independent data set. OC, DFB, LKT, MW, MS, FMV and JH provided the samples. AdR, DSR and ET provided the bioinformatic tools and participated in the analysis. DFB performed the central pathological review. HC and JYD assisted with design of the study and with critical examination of the manuscript. MS and FB conceived of and designed the study, participated in experimental design and interpretation of results and helped edit the manuscript. All authors read and approved the final manuscript.

## Supplementary Material

Additional file 1 **Additional Tables. Table S1**. Patients' clinical characteristics, MGMTP status and classification according to the Phillips and Verhaak classifications. **Table S2**. Centroid-based classifier generating the three groups of gliomas in the present study. **Table S3**. Complete list of minimal common regions and clones with a p-value <0.05 in responders and non-responders to radiation therapy. Genes located in these loci and their corresponding expression p-values are shown. **Table S4**. List of 1000 genes most differentially expressed between responders and non-responders to radiotherapy (500 genes up-regulated in responders and 500 genes up-regulated in non-responders). **Table S5**. List of gene sets with a hypergeometric p-value <0.05 in responders and non-responders to radiotherapy. **Table S6**. Complete list of minimal common regions and clones with a p-value <0.05 in responders and non-responders to first-line chemotherapy. Genes located in these loci and their corresponding expression p-values are shown. **Table S7**. List of 1000 genes most differentially expressed between responders and non-responders to first-line chemotherapy (500 genes up-regulated in responders and 500 genes up-regulated in non-responders). **Table S8**. List of gene sets with a hypergeometric p-value <0.05 in responders and non-responders to first-line chemotherapy. Click here for file

Additional file 2 **Additional methods**. Additional information regarding gene expression and genome data analysis Click here for file
