# Supplementary material for: An ANOCEF genomic and transcriptomic microarray study of the response to radiotherapy or to alkylating first-line chemotherapy in glioblastoma patients
Source: Mol Cancer. 2010 Sep 7;9:234. doi: 10.1186/1476-4598-9-234 (PMC2944185; doi:10.1186/1476-4598-9-234)
Supplement: Additional file 2 — Additional methods. Additional information regarding gene expression and genome data analysis [file 1476-4598-9-234-S2.DOC]

**Addituional Information**

Except when indicated, all transcriptome and genome analysis was carried out using either an assortment of R system software ([http://www.R-project.org](http://www.R-project.org/), V2.10.0) packages including those of Bioconductor (v2.5) by Gentleman et al. [1] or original R code.

**1- Expression microarrays**

We used the *affyQCReport* Bioconductor R package to generate a QC report for all chips. Chips that do not pass this filter were not included in this study.

Raw feature data from the 56 chips were normalized in batch using robust multi-array average (RMA) method by Irizarry et al. [2], implemented in R package *affy*.

**2- CGHarrays**

**2.1- QC, filtering and normalization**

Spots were filtered using the following criteria: (i) signal-to-noise < 2.0 for the reference channel, (ii) control and un-annotated spots (iii) manual flag (iv) raw intensity < 1 (v) raw intensity at saturation (=65000). The remaining raw log2ratio values were normalized using the lowess within-print tip group method from Yang et al. [3] (spots corresponding to X and Y chromosomes were masked to calculate the lowess fit). For clones (BACs/PACs) in which more than 1 feature value remained after filtering and that yielded an inter-feature standard deviation of less than 0.25, an average normalized log2-ratio value was calculated. For each chip the percentage of filtered spots and clones was calculated.

**2.2- Smoothing and breakpoints detection**

The normalized log2-ratio values were smoothed using the tilingArray Bioconductor package v1.24.0 (which implements the method proposed by Franck Picard et al. [4]), yielding smoothed log2-ratios values in homogeneous segments along the chromosome.

**2.3- Copy number assignment**

For each sample, the level (L_N_) corresponding to a normal (i.e. diploid) copy number is determined as the first mode of the distribution of the smoothed log2-ratio values across all -except sexual- chromosomes. The standard deviation (SD) of the difference between normalized and smoothed log2-ratio values is calculated. Then for all clones in a segment, the ‘GNL’ copy number status (G:gain | N:normal | L:loss) is determined as follows, based on the segment smoothed log-ratio value (X): if X > L_N_ + SD then status=gain (G), if X < L_N_ – SD then status=loss (L), else status=normal. Outliers are classed manually and correspond to individual clones that yielded normalized log2-ratio values (Y) such that: Y > L_N_ + 3 × SD (status=gain) or Y < L_N_ - 3 × SD (status=loss).

**2.4- Detection of homozygous deletions and amplicons**

First the rate of tumor cells ‘R_TC_’ was estimated for each sample as 2 - 2^(obsLog2R.1cp+1) were obsLog2R.1cp is the observed median of smoothed log2ratios for clones whose GNL call is ‘Loss’. Then for each sample the expected log2ratio value corresponding to a homozygous deletion was calculated as log2(1 - R_TC_), and this threshold was used on normalized lo2ratios to identify putative homozygous deletions. Putative amplicons were identified the same way using the following threshold : log2(1+ 1.5* R_TC_).

**2.5- Recurrent minimal genomic alterations**

Computation of recurrent minimal genomic alterations was done in a similar way to the method described by Rouveirol et al [5] using original R code.

**3- unsupervised classification of sample expression profiles**

**3.1- Unsupervised probe sets selection**

Probe set’s unsupervised selection was based on the two following criteria:

- a p-value of a variance test (see 3.2) less than 0.01,
- a “robust” coefficient of variation (rCV, see 3.3) less than 10 and superior to a given rCV percentile.

Eight rCV percentile thresholds were used (60%; 70%; 80%; 90%; 95%; 97.5%; 99%; 99.5%) yielding 8 lists of probe sets.

**3.2- Variance test**

For each probe set (P) we tested whether its variance across samples was different from the median of the variances of all the probe sets. The statistic used was ((n-1)×Var(P) / Var_med_), where n refers to the number of samples. This statistic was compared to a percentile of the Chi-square distribution with (n-1) degrees of freedom and yielded a p-value for each probe set. This criteria is the same used in the filtering tool of BRB ArrayTools software [6].

**3.3- Robust coefficient of variation**

For each probe set, the rCV is calculated as follows: having ordered the intensity values of the n samples from min to max, we eliminate the minimum value and the maximum value and calculate the coefficient of variation (CV) for the rest of the values.

**3.4- Obtention of a series of 24 dendrograms**

We performed hierarchical clustering of the samples, using samples profiles restricted to each of the 8 probe sets lists obtained via unsupervised selection (as described above), for 3 different linkage methods (average, complete and Ward’s), using 1-Pearson correlation as a distance metric (package cluster). This analysis produced 24 dendrograms.

**3.5- Stability assessment**

The intrinsic stability of each of the 24 dendograms was assessed by comparing each dendogram to dendograms obtained after data “perturbation” or “resampling” (100 iterations for each). This comparison yielded a mean ‘similarity score’ (see 3.6) across all iterations. Here, perturbation refers to the addition of random gaussian noise (µ = 0, σ = 1.5 × median variance calculated from the data set) to the data matrix, and resampling refers to random substitution of 5% of the samples by virtual sample’s profiles, generated by random crossing of existing profiles.

The overall stability of the classification was assessed by calculating a mean similarity score, using all pairs of dendrograms obtained with the 8 probe sets lists for a given clustering method.

**3.6- Similarity score**

To compare two dendrograms, we compare the two partitions being obtained by cutting in k clusters (k=2..8) these two dendrograms. We then will compare several pairs of partitions (one per value of k). To compare a pair of partitions, we used a similarity measure corresponding to the symmetric difference distance (Robinson and Foulds, 1981).

NB: A similarity matrix A can be obtained from distance a matrix B by posing Aij = X – Bij, where for any pair (i,j), Bij ≤ X.

**3.7- Calculus of a consensus dendrogram and a consensus partition**

To identify the groups of samples that consistently clustered together in the 24 dendrograms (that is robust consensus clusters obtained independently from a given clustering method and/or threshold for unsupervised genes selection), we first calculated a consensus dendrogram using an algorithm proposed by Diday [7] and similar to the approach used by Monti et al. [8]. We proceeded as follows: each of the 24 dendrograms was cut in k clusters, thus yielding 24 partitions for each value of k (k = 2..8). We then calculated the (N,N) symmetrical matrix S of sample’s co-classification (N = number of samples), giving for each pair of samples the number of times they were in the same cluster group in the 24 partitions (from 0 to 24). S is a similarity matrix, and a corresponding distance matrix D is obtained by posing D(i,j) = 24 – S(i,j) (NB: D(i,j) = *maximal_similarity* – S(i,j)). Finally to obtain the consensus dendrogram we use the distance D and complete linkage hierarchical clustering method. Then, cutting this consensus dendrogram in k clusters, we obtained a consensus partition for each value of k (k = 2..8). The level k=3 was choose as it showed very stable clusters.

**3.8- Choice of a representative dendrogram**

To choose a representative dendrogram of the series of 24 dendrograms, we compared each of the 24 corresponding partitions in k=3 clusters to the previously obtained consensus partition in 3 clusters, using the similarity score defined above. Among the dendrograms yielding the highest scores of similarity, we kept the one obtained with the smallest list of genes.

**4- supervised tests and control for multiple-testing**

**4.1- Expression profiles**

Based on the RMA log_2_ single-intensity expression data, we used Limma moderate T-tests (Bioconductor package *limma* [10]) to identify genes differentially expressed. The *p.adjust* function from *stats* R package was used to estimate the FDR using the Benjamini and Hochberg (BH) method [9].

**4.2- Genomic profiles**

Based on the clone based GNL copy number status (-1/0/1), homozygous deletion status (0/1) and amplicon status (0/1), as well as based on MCR alteration status (0/1), we used Fisher exact tests (*fisher.test* function, *stats* R package) to identify clones / MCR with differential status. The *p.adjust* function from *stats* R package was used to estimate the FDR using the Benjamini and Hochberg (BH) method [9].

**4.3- Estimation of the H1 proportion 4.4- Centroids**

Given a series pv of p-values, the related H1 proportion was estimated (using the approach by B. Storey) as (1 – mean{Ind(pv > 0.5)})/0.5, where Ind(x>0.5) = 1 if x>0.5 and 0 otherwise.

**4.4- Centroids**

The centroids classification rule was obtained as follows : we performed an Anova comparing the 3 cluster groups (C1, C2, C3) and retained the probe sets yielding a p-value less than 10-7. For each paired comparison (C1 *vs* C3, C1 *vs* C3 and C2 *vs* C3) we then selected the probe sets corresponding to the 20 highest and 20 lowest geometric mean fold changes, thus obtaining a signature of 62 distinct probe sets (after removing redundancies). Centroids of the 3 cluster groups were calculated as the geometric mean of the probe sets of this signature. Each sample profile (restricted to this signature) was compared to the 3 previously obtained centroids using (1 – Pearson-coefficient-of-correlation) as distance. Samples were attributed to the group yielding the lowest sample to centroid distance.

5- Expression based gene set analyses

**5.1- Gene Sets**

KEGG and Biocarta pathways were respectively obtained from <ftp://ftp.genome.ad.jp/pub/kegg/pathways/hsa> and [http://www.biocarta.com](http://www.biocarta.com/). GO terms and their relationships (parent/child) were downloaded from [http://www.geneontology.org](http://www.geneontology.org/). Molecular Signature Database gene sets and Stanford Microarray Database gene sets were downloaded from <http://www.broadinstitute.org/gsea/msigdb/downloads> and [http://smd.stanford.edu](http://smd.stanford.edu/). We mapped the biological pathway related genes or GO term related proteins to non redundant HUGO gene symbols. For a GO term, we obtained the list of non-redundant related proteins identifiers, either directly associated to the GO term, or to one of its descendants, and mapped it to a non-redundant list of HUGO gene symbols. Mapping between original identifiers and symbols were obtained using *biomaRt* R package.

**5.2- Gene Set Analysis Method**

We used the hypergeometric test to measure the association between a non redundant list of differentially expressed genes and a biological pathway or a gene ontology term (GO term), as in *GOstats* R package. For each comparison (responders vs non responders), 2 lists of differentially expressed genes (limma T-test p<0.05) were analyzed: L_up_ defined as the top 500 most up-regulated probe sets mapped to non redundant symbols, L_down_ defined as the top 500 most down-regulated probe sets mapped to non redundant symbols.

**6- Survival analysis**

Survival curves were calculated according to the Kaplan-Meier method (function *Surv*, R package *survival*, V2.29) and differences between curves were assessed using the log-rank test (function *survdiff*, R package *survival*).

**7- Genome / transcriptome correlation**

We mapped BAC clones and probe sets based on their genomic position. Then for a pair (clone, probe set), subgroups of samples analyzed both for transcriptome and genome were used to calculate a Pearson coefficient of correlation between the normalized log2 RMA intensity values (corresponding to the probe set) and the normalized log2-ratio values (corresponding to the clone).

**REFERENCES**
